# Collectanea Medica

**Published:** 1813-06

**Authors:** 


### COLLECTANEA MEDICA,

CONSISTING OF

ANECDOTES, FACTS, EXTRACTS, ILLUSTRATIONS, QUERIES, SUGGESTIONS, &c.

RELATING TO THE

*History or the Art of Medicine, and the Auxiliary Sciences.*

---

### *On the Liquid Gum from Botany Bay. By* THOMAS THOMSON, M.D. F.R.S.

THE vegetable substance which constitutes the subject of the following experiments was sent to me by Mr. Knox, of Glasgow, under the name of *Liquid Gum from Botany Bay.*

Nothing is known, to me at least, respecting its history, except what the ticket attached to it conveyed. The term *gum*, which is applied to it, seems to indicate that it has exuded spontaneously from some tree or shrub; but the properties of it immediately to be described, render this origin extremely problematical. It is an intermediate substance between the two vegetable principles called *extractive* and *tannin*

*tannin* by chemists. Now no instance, so far as I know, of any species of extractive or tannin having exuded spontaneously from plants can be produced. Indeed, they have all such a tendency to enter into combination, and to form solid compounds with other vegetable principles, that the plant from which any of them could exude spontaneously in a state of purity must be of a very peculiar nature.

The resemblance between this *liquid* gum and kino was so striking as to induce me to examine this last substance. The portion of this drug which is soluble in alcohol is absolutely identical with the solid matter of the liquid gum; but kino contains several substances which are not to be found in our gum. The most remarkable of these is a red substance, which gives a red colour to water, and a still deeper tint to alkaline leys, and which possesses the smell and taste of logwood. It is well known that a part of the kino of the shops comes from Botany Bay as well as our gum; and from the striking similarity between them, I think there is every reason to believe that both of them are obtained from the same vegetable.

The *liquid gum* (for I shall employ that term, though by no means proper, till we have ascertained its properties) has a red colour, approaching to crimson, but considerably darker. It is opake. It has a peculiar smell, and an astringent taste, without the disagreeable intensity of the infusion of nutgalls. Its specific gravity at 60° was only 1·196. Its consistence in the ordinary temperature of the air is nearly the same with that of turpentine. Like that substance, it is very tenacious, and may be drawn out into very long threads. When exposed to cold it becomes harder, and when heated it becomes more and more liquid, till, at the temperature of about 180°, it may be poured from one vessel to another nearly as easily as water. If the phial containing it be plunged into hot water, and kept corked, the whole gum collects at the bottom, without leaving any stain upon the sides of the phial; but in the open air a solid skin almost immediately collects on its surface. On allowing the gum to cool it becomes as thick as at first.

When a portion of the gum is exposed upon a plate of glass to the open air, it gradually becomes solid, contracts in its dimensions, and splits into very regular fragments. The surface of the gum, thus dried, is as smooth as that of sealing wax; the fracture is vitreous, and the colour not unlike that of shell lack. It is very brittle, crumbling to powder between the fingers. The loss sustained amounted to 0·45 of the original weight. On exposing the plate of glass on a furnace to a heat of about 212°, the gum sustained an

 additional additional loss of 0·11 of the original weight; making the whole loss to amount to ·56.

To learn what had occasioned the loss of weight, 100 grains of the liquid gum were put into a small glass matrass, fitted with a capital and receiver, and exposed to a heat not much exceeding 212°. When the distillation was finished, the dry residue in the matrass weighed 43·5 grains. It resembled in every respect the solid matter left when the liquid gum is exposed to the open air. The receiver contained a quantity of water, weighing 53·5 grains. Thus there was a loss occurred amounting to 3 grains. I ascribe this loss to the escape of moisture; for the receiver was not luted.

The water thus obtained was limpid and colourless. It had the peculiar smell of the gum, and a slight taste not easily described. It did not alter the colour of vegetable blues, nor exhibit any thing remarkable, when treated with the different re-agents. The dry residue smelled like bees'-wax, or at least that smell was diffused through the matrass. This dry residue continues always solid and brittle, and is not affected by any degree of heat not sufficient to produce decomposition.

Thus it appears that *our liquid gum* is a compound of 44 parts of solid matter, and 56 parts of water, and that it is indebted to the water for the property of becoming liquid when heated.

To the 43·5 grains of solid residue in the matrass, 56·5 grains of distilled water were added, to make up the original weight. By long-continued digestion in a heat scarcely exceeding 100°, the water gradually incorporated with the dry matter, and formed with it a compound agreeing in every respect with the original *liquid gum*. By distillation this water was driven off: it had the smell of the liquid gum as before, while the dry residue had the odour of bees'-wax.

Here, then, we have a compound to which chemists have not hitherto paid attention, a combination of water and dry vegetable matter, nearly solid while cold; but becoming liquid by heat, like resin or wax.

Though this *liquid gum* contains so great a proportion of water, it exhibits no strong disposition to combine with any more. When put into water it immediately tinges that liquid of a muddy red, but a portion falls to the bottom; nor was I able to procure a complete solution of it in cold water. Hot water acts better; but a portion precipitates again as the solution cools.

The aqueous solution remains muddy, even after standing for weeks; but by repeated filtrations, or by mixing it with hot water, it may be obtained transparent.

When

When a drop of water is let fall upon the liquid gum, the spot on which it lights becomes lighter coloured, as if the solid matter consisted of two distinct substances; one, dark coloured, more soluble in water; and the other light coloured, and less soluble: but I found it impossible to procure any two such substances in a separate state, either by means of water, or any other re-agent.

The aqueous solution of our liquid gum is of a dark red colour inclining to brown; its taste is the same with that of the gum; and when evaporated to dryness, it leaves behind it a substance precisely the same as the solid residue procured by exposing the liquid gum to the open air.

Alcohol is the proper solvent of our liquid gum; taking it up very readily, both in its natural state, and when deprived of its water by evaporation: but the solution takes place much more readily in the first than in the second state.

Alcohol of the specific gravity of 0·800 easily takes up half its weight of the gum. The solution is blood-coloured, and so deep as to be opake; but a drop or two put into a phial of alcohol gives the whole a beautiful crimson colour.

The solution has an astringent taste, and may be diluted with water without any precipitation. Indeed, the addition of alcohol to the muddy aqueous solution makes it transparent, and prevents all subsidence.

3. Sulphuric ether is slightly coloured by the gum, but dissolves only a very small portion of it.

Thus it appears that the so called liquid gum is a compound of 44 parts solid matter and 56 water; that it is soluble both in water and alcohol, and weak spirits. It remains for us to ascertain the properties of the solid matter. For this purpose I exposed the aqueous solution to a variety of chemical agents. The following are the most important facts.

### *Action of Glue.*

When a solution of glue is mixed with the aqueous solution of our gum, the mixture becomes immediately filled with flesh-coloured flashes, which gradually subside, leaving the solution colourless. If the glue has been cautiously added, as long as any precipitate fall the whole gummy matter of the so called gum is separated; for when the liquid is evaporated to dryness, nothing is obtained but a little glue. When the flesh-coloured precipitate thus obtained is dried, it becomes brown, and bears a strong resemblance to the precipitate thrown down from catechu or nutgalls, by glue.

glue. Its colour is lighter, but it possesses all the chemical characters of that precipitate.

If a bit of fresh skin be steeped for some weeks in our aqueous solution, it is converted into leather, precisely as when steeped in the infusion of oak bark.

Hence it follows that the solid matter of our gum possesses the essential character of *tannin.*

### *Action of the Metalline Salts.*

All the metallic solutions which I tried occasioned precipitates when dropped into the aqueous solution of the gum; but none of them rendered the solution colourless, except nitrate of mercury. The following table exhibits the colour of the precipitate, and of the supernatant liquid:

| | Precipitate. | Liquid. |
|---|---|---|
| Nitrate of mercury | Reddish brown | Colourless. |
| Corrosive sublimate | O | Muddy, and light-coloured. |
| Sulphate of copper | Ochre yellow | Brown. |
| Sulphate of zinc | Light brown | Brown. |
| Sulphate of iron | Brown black | Dark green. |
| Nitrate of silver | Dark grey | Grey brown. |
| Muriate of nickel | Light yellow | Light yellow. |
| Muriate of arsenic | Ditto | Ditto. |
| Muriate of manganese | Grey brown | Grey brown. |
| Cuprate of ammonia | Dark brown | Dark brown. |

These precipitates, when dried, assumed all of them a colour more or less brown, none of which answered well as paints, at least when used with water. The whole of the colouring matter could not be thrown down by these salts. Hence it follows that they form with it compounds to a certain degree soluble in water.

### *Action of the Earths.*

The three soluble alkaline earths, barytes, strontian, and lime, when dropped into the solution, throw down a beautiful dark brown precipitate in flocks; but the solution still continues coloured, though an excess of the earthy waters be added. When the precipitate is dried it retains its colour, is tasteless, but gives a brown colour to water.

Muriate of alumina likewise renders the solution brown and opake, and a precipitate falls after some hours, which does not appear to be soluble in water. Neither muriate of magnesia, nor muriate of barytes, occasions any change; neither is any precipitate thrown down by silicated potash.

The

### *Alkalies.*

The pure alkalies render the liquid very dark coloured and opake, so that it appears black, though in reality it is only of a very deep brown. The compound does not precipitate with glue, at least immediately.

The liquid sulphuret of potash occasions a copious yellow-coloured precipitate.

When an acid is added to the alkaline solution, the original colour is gradually restored, and the liquid becomes again transparent.

### *Acids.*

The strong acids, namely, the sulphuric, nitric, and muriatic, immediately precipitate a yellow or brown powder, and the liquid retains its deep brown colour.

Oxymuriatic acid immediately destroys the colour, renders the solution transparent, and throws down yellow flakes. The colour is not again restored by adding an alkali.

The tan was destroyed; for the solution being evaporated, yielded only muriate of manganese, sulphate of lime, and little brown extractive insoluble in water.

### *Nature of the Solid Matter.*

The properties above detailed are sufficient to show us that the substance which constitutes the essential part of our gum lies intermediate between extractive and tannin, as they have been described by chemists.

It agrees with tan in being thrown down by glue, in converting skin into leather, and in precipitating most of the metallic salts; but there are two species of tan, differing essentially from each other in many respects.

The first, for the sake of distinction, may be called the tannin of nutgalls, or of oak bark. It is insoluble in alcohol, forms a brown precipitate with glue, and a black with iron. Water is its best solvent. In all these particulars our tannin differs from it.

The second species, which has been discovered by Hatchett, may be called artificial tannin. It is soluble in alcohol, and throws down iron and glue brown. Its taste is astringent and bitter. Our tannin agrees more nearly with this species than the preceding, though the differences are also considerable. Nitric acid does not injure artificial tannin, but it converts the tannin of liquid gum to the bitter principle of Welter.

Extractive is distinguished by being soluble in water, and becoming insoluble by exposure to the air, but continuing soluble soluble in alcohol. It is thrown down in yellow flocks by oxymuriatic acid: it dies cotton brown. In all these respects the solid matter of liquid gum agrees with it.

Chemists have not hitherto perceived that extractive and tannin of nutgalls stand at the two extremes of a series of substances which gradually pass into each other, and that they are so closely connected that they might all be not improperly arranged under one genus, and receive a common name. Indeed, till this is done, the confusion which at present subsists in this department of vegetable chemistry, cannot easily be removed.

When nitric acid is poured upon the solid matter of liquid gum, it softens and dissolves it with the evolution of a little nitrous gas. The solution has a very dark red colour; but, on being heated, that colour rapidly disappears, and the solution becomes a very light yellow. When distilled, it diffuses the smell of prussic acid; by continuing the distillation to dryness in a very moderate heat, a solid matter is obtained, white if the heat has been a minimum, yellow if a little higher, and brown if the fire has been urged too far. This matter is soft and spongy. It has an intensely bitter taste, readily dissolves in water and alcohol, melts when heated, without undergoing any other change. When the solution of this matter in water is evaporated, small crystals are formed, which detonate when heated. This matter is similar to what has been called the bitter principle of Welter. It is best procured by treating indigo with nitric acid. Its properties have been detailed at full length by Hatchett, Fourcroy, and Vauquelin.

When the tanno-extractive of our gum is exposed to heat, it swells, blackens, and melts, and a considerable portion of gas is evolved. The first portion of gas is attended with a dense white vapor, and is absorbed by water so rapidly, that I have little doubt that it is ammonia, though I had too little of the gum to be able to verify this supposition by repetition of the experiment. After this, the gas obtained is carbonic acid, and carbureted hydrogen. Water comes into the receiver, which is strongly impregnated with a portion of tanno-extractive, not much altered. The charcoal that remains burns to ashes as easily as tinder. The ash that remains is very trifling. It consists, as far as I can judge, of sulphate of lime and carbonate of lime; but other earths might easily have escaped my detection, for the quantity of ashes which I examined did not exceed the hundredth part of a grain.

From the preceding detail, it is obvious that the substance which which we have been examining is a combination of a variety of tannin and water, and that it has no resemblance whatever to gum.

---

*The Description of an Organ by which the Eyes of Birds are accommodated to the different Distances of Objects. By* PHILIP CRAMPTON, Esq.

In the philosophical investigation of the phenomena of vision, the eye must be considered under two aspects—as an optical instrument, and as an organ of sense. This mixed nature of the organ subjects the analysis of its functions, upon mathematical principles, to considerable uncertainty; for the perceptions of sight must be considered as depending upon certain motions or affections of the nervous power, which, although originally excited by the agency of light, may exist or be renewed without its presence. In delirium, for example, the perception of objects that have no existence is as perfect as if their images were formed by rays accurately converging upon the retina. In addition to this, it may be observed, that the eye is not a perfectly achromatic instrument, yet objects are not seen with that kind of indistinctness which might be supposed to result from this imperfection in its optical constitution. It appears, then, that in the present state of our knowledge, it must be difficult, if not impossible, to ascertain how much, if any, apparently optical effect is to be attributed to the mechanical constitution of the eye, and how much to the agency of the principle of life. This cannot be better illustrated than by referring to the contradictory opinions which have been maintained among the most distinguished natural philosophers, with respect to the faculty which the eye is thought to possess of adjusting its focus to the different distances of objects. All the hypotheses which have been framed to explain the means by which this adjustment is effected, have proceeded upon the supposition that it was necessarily connected with some change, either in the external configuration of the eye, or in the relative position of its internal parts. Now, although it is certain that to form a *perfectly* distinct image upon the retina, the focus of the eye must be accommodated to the distance of the object, still we have no proof that such a *perfect* image is essential to distinct vision.

The nervous influence, whatever may be its nature, which conveys to the sensorium a knowledge of those properties of bodies which are the proper objects of vision, may be as perfectly excited by rays possessing one degree of convergency as another; for we cannot entertain the gross conception of the mind sitting behind the eye to contemplate the pictures which are painted upon the retina. The impressions of light and colors upon the eye, like the impressions of articulate sounds upon the ear, suggest the things which they signify, not by any likeness or identity of nature, but only by an habitual connection, which constant experience has proved to subsist between them; nor is the supposition which attributes to the eye the faculty of seeing objects distinctly by rays which do not accurately converge upon the retina, altogether without foundation, since M. de la Hire* has demonstrated that "the eye does not change its conformation, or adapt itself to the distances of objects, when they are viewed through a perforated card." For if a small object, placed at that distance from the eye at which vision is most distinct, be viewed through three pin-holes, so disposed that the interval between the most distant of them shall not exceed the diameter of the pupil, the object will be seen single; but if the object be brought either within or beyond the limits of distinct vision, it will be seen multiplied as many times as there are holes in the card, and each of the three images will be as perfect as the single one. It is obvious that the three images are formed by three pencils of rays, which are cut by the retina, either before their convergence or after their decussation, and consequently the object is seen distinctly by rays which do not accurately converge upon the retina. Now, although there is every reason to distrust M. de la Hire's general conclusion, that the eye is not adapted to the different distances of objects by any change in its optical conformation, still I believe it will be readily admitted, that, if the reality of such a change be at all questionable, the hypotheses which have been contrived to explain the means by which it is effected, cannot be received as proofs of its existence. Sir Everard Home and Mr. Ramsden, in the true spirit of philosophical research, endeavored to bring the matter to the test of experiment; but the only conclusion which those philosophers felt themselves authorised to draw from experiments conducted with a degree of accuracy of which the subject seemed scarcely susceptible, was, "that a change in the length of the axis of vision, for the purpose of adjusting the eye, is rendered highly probable;"† but since, if it happened at all, it could not exceed $\frac{1}{100}$th part of an inch, and as such a change would not account for the phenomena, it became necessary to introduce

* M. de la Hire, Journal des Sçavans, 1693. Porterfield on the internal motions of the eye.

† Croonian Lecture, Phil. Trans, 1796.

the the agency of other causes, which, as they could not be subjected to the test of experiment, must be considered as merely hypothetical. The strongest argument in favor of the internal changes of the eye seems to be drawn from comparative anatomy; for if it be true that there is in the eyes of birds an organ which regulates the focal distance of the crystalline lens, then (resting upon the uniformity in nature's laws) we may be assured that in *all* animals the eye is accommodated to the different distances of objects by some change in its optical constitution, although the precise nature of that change, or the means by which it is effected in the various classes of animals, may for ever elude our research. I believe Derham* first entertained the opinion which ascribes to the marsupium, or pecten plicatum, in the eyes of birds, the function of regulating the focal distance of the eye. The opinion has lately been revived by Sir Everard Home, but it seems liable to insuperable objections; for, 1st, the marsupium does not exhibit the least trace of muscular structure. I have very carefully examined this organ in the ostrich and the eagle: in these great birds, the part is of such a size that were it really muscular the fibrous structure could scarcely escape detection.†

2d. In many birds, as in the turkey, jackdaw, &c. the marsupium terminates in the substance of the vitreous humor, and has no direct attachment to the lens."‡

3d. In all birds it is situated obliquely with respect to the lens, so that if it acted at all, it could merely communicate to the lens a motion of rotation, or remove it a little from the axis of vision: this cannot be better illustrated than by referring to Sir Everard Home's excellent representation of this organ (Phil. Trans. 1796). It would seem, then, that we are ignorant not only of the means by which the optical constitution of the eye is so changed as to be accommodated to the different distances of objects, but that we have not hitherto had satisfactory evidence that any such change takes place.

Such I believed to be the state of the subject, when, in the month of February last, an opportunity occurred to me of examining the eye of an eagle, and shortly after that of an ostrich. In those great birds we can view all the peculiarities which distinguish organs of vision in the feathered

* Physico-Theol. note to p. 106.

† In the ostrich the marsupium measured $\frac{8}{10}$ of an inch from the apex to the base, and the longest diameter of the base itself measured $\frac{7}{10}$ of an inch.

‡ Blumenbach's Comparative Anatomy.

tribe in general, rendered large and conspicuous. To this circumstance I am indebted for the discovery of an organ, which I trust will enable us to solve a problem in optical science, which has long occupied the attention of some of the most distinguished members of the Royal Society.

The organ to which I allude is a distinct muscle, which arises from the internal surface of the bony hoop of the sclerotica, and is inserted by a tendinous ring into the internal surface of the cornea, about one line within its circumference. In order to demonstrate this muscle, it is necessary only to remove the anterior segment of the eye just behind the bony hoop, and then the pigmentum nigrum being carefully washed away, the iris is to be gently detached from the ciliary circle, and the choroid coat from the sclerotica. Some delicacy is necessary in performing this part of the operation, for the muscular fibres adhere to the internal surface of the choroid coat, as well as to the bony hoop: if the choroid, therefore, be not slowly and carefully detached, many of the muscular fibres will be separated from the bone, and confounded with the membrane and its pigment.

When the muscle is thus exposed, its descending fibres will be seen terminated in a well-defined tendinous ring, which advances a little beyond the circumference of the cornea, to which it firmly adheres. The thickness of the muscle, as well as the manner of its insertion, may be most conveniently demonstrated by cutting the anterior segment of the eye through its diameter. The fibres will then be seen upon that part of the cut edge which corresponds with the bony hoop. To complete the demonstration, a pin, or thin probe, may be passed between the muscle and the sclerotica. The nerves, which are seen branching in a singularly beautiful manner through the substance of the muscle, are derived from the lenticular ganglion; a mere inspection of the attachments of this muscle will be sufficient to suggest its action; for since the bony hoop, from which the fibres arise, must be considered as a fixed point, the cornea into which they are inserted must be drawn inwards by their contraction; but the matter admits of demonstration. By means of the galvanic influence, the action of the muscle may be excited in the eye of a turkey a few minutes after the head has been separated from the body, when it may be observed that every contraction of the fibres is attended with a corresponding motion of the cornea; or if the fibres be drawn upwards by means of a forceps, the cornea may not only be flattened, but its convexity may be made to *respect* the iris. Since, then, it may be demonstrated that this muscle is in its action a depressor of the cornea, it seems scarcely necessary to add that its influence must tend to diminish the convexity of the eye. It seems probable, therefore, that the eyes of birds are, in the ordinary state, possessed of a high refractive power; and an eye so constituted, seems peculiarly well adapted to the uses of the animal while it rests upon the earth, but when it soars into the middle regions of the air, the rays proceeding from objects below must arrive at the eye in lines which may be considered as parallel; consequently, to form any thing like a distinct image, the focal length of the organ must be increased as the divergency of the rays decreases. This adjustment may be perfectly effected by diminishing the convexity of the cornea, and it has been shewn that there is in the eye a muscle to which this function may be assigned.

